# First record and morphological characterization of an established population of *Aedes* (*Hulecoeteomyia*) *koreicus* (Diptera: Culicidae) in Germany

**DOI:** 10.1186/s13071-018-3199-4

**Published:** 2018-12-17

**Authors:** Wolf Peter Pfitzner, Alice Lehner, Daniel Hoffmann, Christina Czajka, Norbert Becker

**Affiliations:** Kommunale Aktionsgemeinschaft zur Bekämpfung der Schnakenplage e. V. (KABS), Georg-Peter-Süß-Str. 3, 67346 Speyer, Germany

**Keywords:** *Aedes koreicus*, *Aedes japonicus*, Germany, Distribution, Morphological comparison, *nad*4

## Abstract

**Background:**

The East Asian mosquito species *Aedes koreicus* was recorded out of its native range for the first time in Belgium in 2008. Since then, several other European populations or single individuals have been observed throughout Europe with reports from Italy, Switzerland, European Russia, Slovenia, Germany and Hungary. The Italian population seems to be the only one that is expanding rapidly, so the Swiss population very likely derives from it.

**Results:**

In a surveillance program for invasive mosquito species, a single larva of *Ae. koreicus* was found in a cemetery vase in 2016 in the city of Wiesbaden, Germany. In the following year the finding was confirmed and an established population could be proven over an area of about 50 km^2^. The morphological identification of the first larva was confirmed by sequencing of a region within the *nad*4 sequence. A study of adult females showed that the morphological characteristics of this population are not identical to the populations from Belgium and Italy. The eggs and larvae were found together with *Aedes j. japonicus* in the same breeding sites and ovitraps, as well as with other indigenous mosquito species such as *Culex pipiens*/*Culex torrentium*, *Aedes geniculatus* and *Anopheles plumbeus*.

**Conclusions:**

Since the newly discovered population in Germany shows different morphological characteristics to the populations in Belgium and Italy, it seems to originate from an independent introduction. It remains unknown how the introduction took place. A further spread similar to the one in northern Italy can be assumed for the future due to similar climatic conditions.

**Electronic supplementary material:**

The online version of this article (10.1186/s13071-018-3199-4) contains supplementary material, which is available to authorized users.

## Background

The container-breeding mosquito species *Aedes* (*Hulecoeteomyia*) *koreicus* (Edwards) is native to Korea, north-eastern China and eastern Russia [1]. It appears to be quite uncommon in Japan and was reported by LaCasse & Yamaguti [2] only from the north of Hokkaido. It is closely related to the Asian bush mosquito *Aedes* (*Hulecoeteomyia*) *japonicus japonicus* (Theobald), which has spread over North America and central Europe in recent years [3]. *Aedes koreicus* is supposed to have a similar potential for becoming introduced to remote countries [4]. Indeed, this species was first discovered out of its native range in 2008 in Belgium, where it became successfully established [5]. It is distributed over an area of only a few square kilometers and does not seem to be spreading. A second European population was found in 2011 in Italy, in Belluno Province in the north-east at the edge of the Alps [6]. In 2013, *Ae. koreicus* was shown to be present over an area of about 3000 km^2^ [7]. The path of introduction could not be determined and it is assumed that the species had already been present for several years. Up to 2016, it had expanded its distribution mainly into southern and western directions [8, 9]. There were also findings in some remote areas in north-western Italy, which correspond to new records of the species in the Swiss-Italian border region in 2013 [10]. This shows that *Ae. koreicus* is spreading quite dramatically in northern Italy, comparable to the expansion of *Ae. j. japonicus* in Germany [11, 12] (B. Pluskota, unpublished observations) or in Austria [13]. This fact stands in contrast to Belgium, where the population seems to remain restricted to the primarily colonized area in an industrial zone [14]. Other reports of *Ae. koreicus* in Europe come from Russia [15], Slovenia [16], Germany [17] and Hungary [18].

Little is known about the vectorial potential of *Ae. koreicus*. Japanese encephalitis virus was found in field-caught specimens [19] and females can be infected with chikungunya virus [20] and microfilariae of the dog heartworm *Dirofilaria immitis* [7] by feeding on infected blood in the laboratory.

*Aedes koreicus* seems to form a monophyletic group together with the four subspecies of *Ae. japonicus* [4] and shows a high morphological similarity to them in the adult stage. Males cannot be distinguished morphologically, and all specific characteristics of the females show a degree of overlap among species [1]. The first record of *Ae. j. japonicus* in Germany was in 2008 in the south-west on the border with Switzerland [21, 22]. Since then, it has become widely distributed over the whole federal state of Baden-Württemberg [11, 23] and to neighboring federal states (B. Pluskota, unpublished observations). There are other independent but smaller populations in western, northern and south-eastern Germany [12, 24]. The species can be regarded as established and its distribution has also expanded to the area where the Kommunale Aktionsgemeinschaft zur Bekämpfung der Schnakenplage (KABS) is conducting a mosquito control program along the River Rhine, mainly targeting floodwater mosquitoes, but also *Culex pipiens* biotype *molestus* (Forskal) and *Anopheles plumbeus* (Stephens) in the townships. In 2013, a surveillance program was initiated to check water containers in the cemeteries of the KABS member municipalities for the presence of *Ae. j. japonicus*.

Within this program, a single larva of *Ae. koreicus* was found in 2016 in Wiesbaden, a city in the northern part of the area. In the following year, a survey was started to validate this finding and to evaluate the establishment and distribution of the population. Additionally, a detailed morphological study was performed for the discrimination of the species to *Ae. j. japonicus* and to identify the morphological characteristics and therefore the presumptive origin of the population.

## Methods

### Larvae collection

**Fig. 1:**
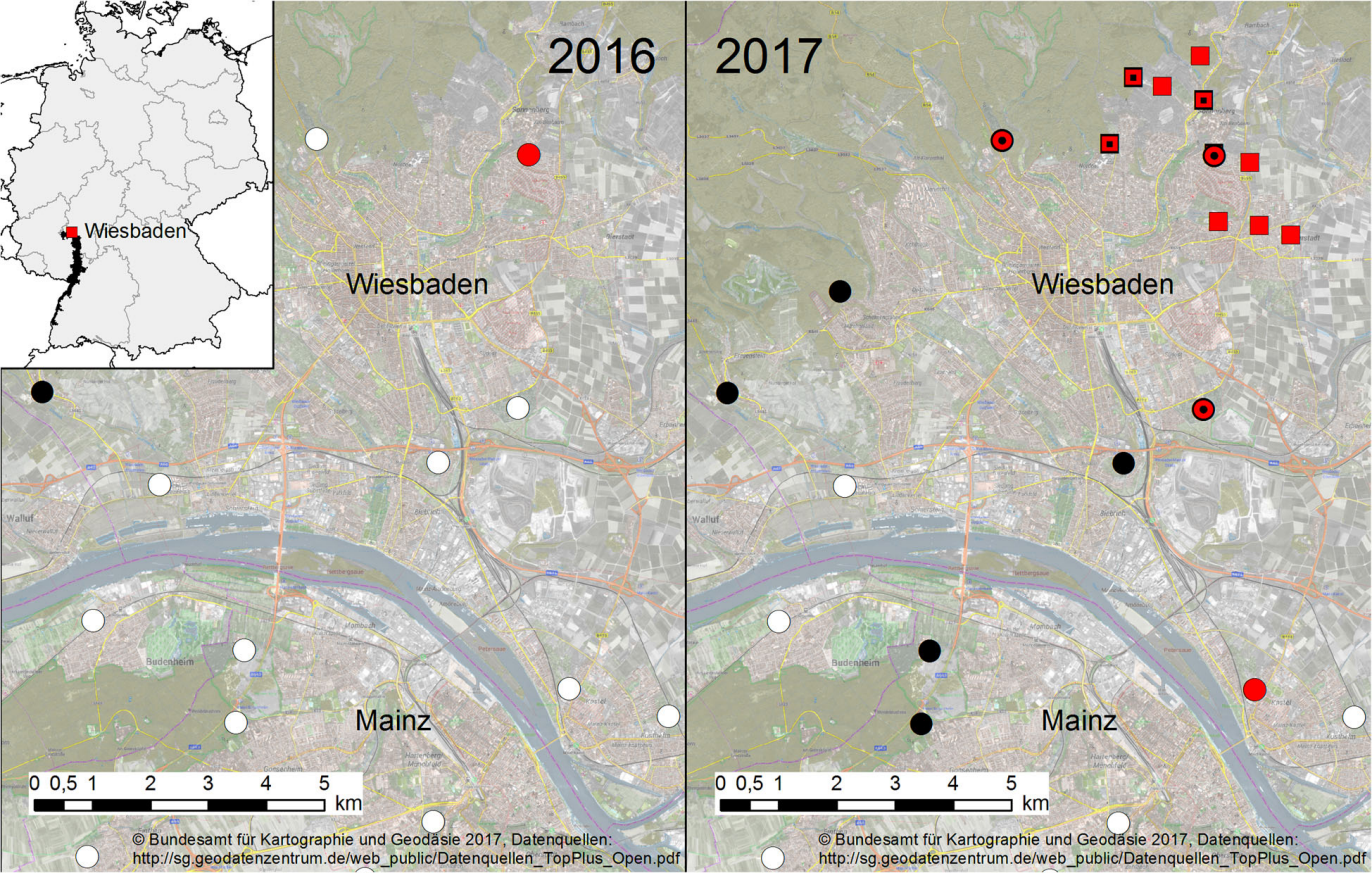
Map of ovitraps and sampled cemeteries in Wiesbaden and Mainz with results for *Ae. koreicus* and *Ae. j. japonicus* in 2016 and 2017. Location of Wiesbaden in Germany in overview with KABS municipalities in black. Circles, cemeteries; squares, ovitraps; white, negative for both species; red, positive for *Ae. koreicus*; black, positive for *Ae. j. japonicus*; red with black dots inside, positive for both species. Includes material © 2018 Planet Labs Germany GmbH. All rights reserved. Data provided on behalf of the German Aerospace Center through funding of the German Federal Ministry for Economic Affairs and Energy

In 2013, the KABS started a surveillance program for the early detection of invasive mosquito species, primarily *Ae. j. japonicus*. Within this framework, cemeteries of the KABS member municipalities were investigated once a year and all larvae found in cemetery vases and water basins were collected and stored in glass vessels containing 50 ml of 70% ethanol. The collections usually took place in late August or early September, depending on rainfall and temperature which influence the hatching and speed of development. The larvae were morphologically identified in the laboratory using a stereomicroscope with a magnification up to 100×, following the keys of Becker et al. [25], Mohrig [26] and Tanaka et al. [1]. The KABS member municipalities are situated in south-western Germany along approximately 300 km of the Upper Rhine, ranging from the mountain Kaiserstuhl near Freiburg (about 60 km north of the Swiss city Basel) in the south, up to the city of Bingen in the north where the Rhine starts running through the Rhine gorge (Fig. 1).

During this surveillance program, a single larva collected from a cemetery in the city of Wiesbaden in September 2016 was morphologically identified as *Ae. koreicus* using the descriptions by Tanaka [1] and Versteirt [5]. The larva was mounted on a slide fixed in Euparal (Carl Roth GmbH, Karlsruhe, Germany) and additionally compared to larvae from Belgium treated in the same way. Abdominal segments III–V were cut off before preparation for further molecular analyses.

After this detection, a survey was started in Wiesbaden the following year in order to investigate whether the larva was part of an established population and to evaluate the actual distribution of the species. The city is situated in the Rhine-Main area, about 20 km west of Frankfurt/Main, next to the river Rhine and bound by the low mountain range High Taunus in the north-west and the mouth of the River Main in the south-east. It has a temperate climate with an annual mean temperature of 10.4 °C and an average annual precipitation of 653 mm (meteorological station Wiesbaden-Süd, 147 m, 1981–2010) [27, 28]. The elevation ranges between 83 and 608 m in the bordering mountains.

### Ovitrap collection

The first larva of *Ae. koreicus* was collected in the Sonnenberg District, located north-east of the city center; therefore this point was chosen as the center of the investigation area. Nine sampling sites were determined within a radius of 2 km, including two cemeteries, three locations in the forest, three private gardens and one industrial area to find the range of the infested area in the surroundings of the first detection. The elevation of these sites ranges between 180–260 m.

Between one and five ovitraps were placed at each site, depending on the size and environmental conditions. Locations near bushes and hedges hidden from passers-by and also allowing rainwater to refill the traps were preferred. The traps were assembled from black plastic trays (25 × 18 × 14 cm) (Regalux Clear Box XXS, Bauhaus, Mannheim, Germany) with brown clay flowerpots (17 × 14 cm diameter) as oviposition substrate which were placed upside down inside the trays. Initially the traps were filled with 2 l of tap water and protected from overflowing by a hole 4 cm below the rim at each short side. During the investigation period the water levels were maintained by both rainwater and manual refilling when sampling the traps.

Ovitraps were collected three times between July and October 2017. Eggs were removed from the flower pots with a soft brush and put into 500 ml of tap water for hatching. Hatched larvae as well as the larvae directly collected from the ovitraps were reared to the fourth-instar, killed and stored in 70% ethanol until identification and counting of the larvae. About 100 individuals from five different sampling sites were reared to adult stage for morphological investigation of the females. Cemetery vases and water basins at the Sonnenberg cemetery were monitored twice, on July 4 and August 14.

Additionally, the routine surveillance program was continued in 8 other cemeteries in Wiesbaden. The cemeteries were sampled once at the beginning of September. Their elevation ranged between 88–230 m.

### Molecular identification

The larva collected in 2016 was identified *via* the sequencing of a fragment of the mitochondrial *nad*4 gene in addition to morphological identification. DNA extraction (QuickExtract Extraction Solution, Epicentre, Madison, WI, USA) was performed according to the manufacturer’s protocol and the extracted DNA was kept frozen at -20 °C until further use.

A region within the *nad*4 sequence was amplified by PCR [29] using the following reagents: 5× Q5 OneTaq Standard Reaction Buffer (New England BioLabs, Ipswich, MA, USA; final concentration: 1×), 1.25 U of OneTaq Hot Start DNA Polymerase (New England BioLabs), 200 μM each of dNTP (‘dNTP-Mix 25 mM’, Biozym, Hessisch Oldendorf, Germany) and the primers N4J-8502D and N4N-8944D in final concentrations of 0.3 μM.

An initial denaturation (30 s at 94 °C) was followed by 35 cycles of denaturation at 94 °C for 15 s, annealing at 53 °C for 45 s and elongation at 68 °C for 60 s. The cycling ended with a final elongation step at 68 °C for 5 min.

After checking the size of the PCR product *via* agarose gel electrophoresis (2%, pre-stained with MidoriGreen, Biozym) the amplicon was prepared for sequencing using the Monarch PCR & DNA cleanup-kit (New England BioLabs) following the manufacturer’s protocol. Sequencing was carried out by Eurofins Genomics (Ebersberg, Germany).

### Morphological study

Females were studied to determine the morphological characteristics of the introduced specimens. In Belgium and Italy the morphological variant from the Korean island Jeju-do was reported [5, 6], which differs from the Korean mainland population. The characters examined, as given by Tanaka et al. [1] and Versteirt et al. [5], are described in Table 1. These are the color of pedicel scales and erect fork scales on the vertex, the scales on the antepronotum and the pale basal bands on the hind tarsomeres. Versteirt et al. [5] describe the last of these as the main difference. While the hind tarsomere V bears a clear basal band in the Belgian and Jeju-do population, it is usually completely dark in the Korean mainland population and occasionally displays an incomplete pale basal band.

**Table 1:** Examined morphological characters of female *Ae. koreicus* and *Ae. j. japonicus*, following the descriptions of Tanaka et al. [1] and Versteirt et al. [5]

	*Ae. koreicus* mainland	*Ae. koreicus* Jeju-do	*Ae. j. japonicus*
Differentiation of *Ae. koreicus* from mainland Korea and Jeju-do island (Tanaka)			
Fork scales	Entirely dark, max. 4 pale scales	With pale scales (1–3, up to 10)	Often entirely dark
Antepronotum	Broad pale scales	Pale falcate scales	Broad pale scales, occasionally few falcate scales
Differentiation of *Ae. koreicus* from mainland Korea and Jeju-do island and *Ae. j. japonicus* (Tanaka/Versteirt)			
Pedicel scales	More pale than dark scales	Dark, dorsal and lateral pale spots	More dark than pale scales
Hind tarsomere IV	Pale basal band	Pale basal band	Usually dark
Hind tarsomere V	Usually dark	Pale basal band	Usually dark
Differentiation of *Ae. koreicus* and *Ae. j. japonicus* (Tanaka)			
Postpronotum	Usually with dark scales	Usually with dark scales	Usually without dark scales
Subspiracular patch	Present	Present	Absent
Costa	Entirely dark	Entirely dark	Ventrobasal pale or grey scales
Hind femur	Base entirely pale	Base entirely pale	Dark subbasal band

Another examined difference was the characteristic pattern of lines on the scutum, which had a silver-white color in the Belgian population, whereas all Korean individuals showed yellowish-brown or golden-yellow scales. Although the pattern of pale scales on the abdominal terga revealed a high degree of variation, the Jeju-do and Belgian specimens sometimes missed the basolateral patches, whereas the basomedian patches were usually very thin [5].

Since there seem to be some uncertainties in the discrimination of the two closely related invasive species *Ae. koreicus* and *Ae. japonicus*, additional characters were examined comparatively using individuals of *Ae. j. japonicus* collected in Wiesbaden and other locations in the range of the south-west German population (see next section). These are the scutal pattern [30], the scales on the postpronotum, the subspiracular patch, which is usually absent in *Ae. j. japonicus*, the presence of pale scales on the costa and the coloration of the hindfemur [1] (see Table 1). Two features mentioned before, the color of the pedicel scales and the pale basal bands on hindtarsomeres IV, are also reported as specific characteristics [1]. Females were studied using a stereomicroscope with a magnification of 100×.

### Origin of examined individuals

Altogether, 43 females of *Ae. koreicus* were studied for morphological characteristics. They originated from five different ovitrap sites in Wiesbaden and were derived by rearing the larvae to the adult stage; they numbered 3 to 23 individuals per site. Thirty specimens of *Ae. j. japonicus* were examined from six different sites. One site was located in Wiesbaden where the larvae were collected from an ovitrap and reared to adult stage. The other individuals were sampled from a cemetery vase in the southern Upper Rhine Valley, two different rock pool locations in the Black Forest and two locations on the western edge of the Upper Rhine Valley in the Palatinate region from a cemetery vase and a stone tray. The numbers of the collected specimens ranged from two to eight per site. The females were identified following the key of Tanaka et al. [1] before the detailed examination of the specific characters.

## Results

### Ovitrap and larvae collection

All nine ovitrap sampling sites within the 2 km radius around the Sonnenberg cemetery in Wiesbaden were found to be positive for eggs and larval stages of *Ae. koreicus* on 1 to 3 sampling dates (Fig. 1). At four ovitrap sites, *Ae. j. japonicus* could also be found, while *Culex pipiens* L. or *Culex torrentium* (Martini) was present at all nine sites. Vases and water basins in the Sonnenberg cemetery were positive for larvae of *Ae. koreicus*, *Ae. j. japonicus*, *Cx. pipiens*/*Cx. torrentium* and *An. plumbeus*. In the ovitraps, numbers of larvae of *Ae. koreicus* ranged between 3–131 with an average number of 50 larvae per positive trap (mean = 49.7, SD = 40.5). Altogether, 16 of the 27 ovitrap samples were positive for *Ae. koreicus*. In these traps, the species was associated with *Ae. j. japonicus* twice, with *Cx. pipiens*/*Cx. torrentium* nine times and with both species four times. On both sampling dates in August, 7 ovitraps were positive for *Ae. koreicus* whereas in October only 2 ovitraps were positive, both of which had been found positive before. The ovitraps in the Sonnenberg cemetery were positive on all three sampling dates (see Additional file 1: Table S1).

On August 14, the number of larvae and the species composition was evaluated in each vase individually in the Sonnenberg cemetery. Only 1 vase out of 62 was positive for *Ae. koreicus*, 1 for *Ae. j. japonicus*, 26 for *Cx. pipiens*/*torrentium* and 6 for *An. plumbeus*. *Aedes koreicus* was found together with *An. plumbeus*, and *Ae. j. japonicus* together with *Cx. pipiens*/*Cx. torrentium* and *An. plumbeus*.

Ongoing sampling in the framework of the KABS surveillance program in Wiesbaden outside of the 2 km radius showed 3 more cemeteries to be positive for *Ae. koreicus*. *Aedes j. japonicus* was detected in 5 out of 8 additionally sampled cemeteries (Fig. 1). *Culex pipiens*/*Cx. torrentium* occurred in all of the cemetery locations. Larvae of *Aedes* (*Dahliana*) *geniculatus* (Oliver) (3/8) and *An. plumbeus* (6/8) were also found. In 2 cemetery sampling sites, all 5 species were present. In the city of Mainz south of the Rhine, *Ae. j. japonicus* was found in 5 of the 12 cemeteries screened in 2017. All 12 cemeteries were negative for invasive mosquito species (IMS) in the year before.

### Molecular identification

The sequencing of the amplified *nad*4 region resulted in a sequence of 381 bp (GenBank: MK069483). The output of the BLAST search showed 99% identity with sample sequences of *Ae. koreicus* from Germany (KT962063) and Belgium (JF430392), 100% similarity with a shorter sequence (333 bp) from Belgium (KJ623735) and 99% identity with shorter sequences from Belgium (310–344 bp; KJ623732-KJ623734) and from Korea (327 bp; GU229925-GU229927). The closest similarity to *Ae. j. japonicus* was 92% similarity to a sample sequence from USA (DQ470164).

### Morphological study

**Fig. 2:**
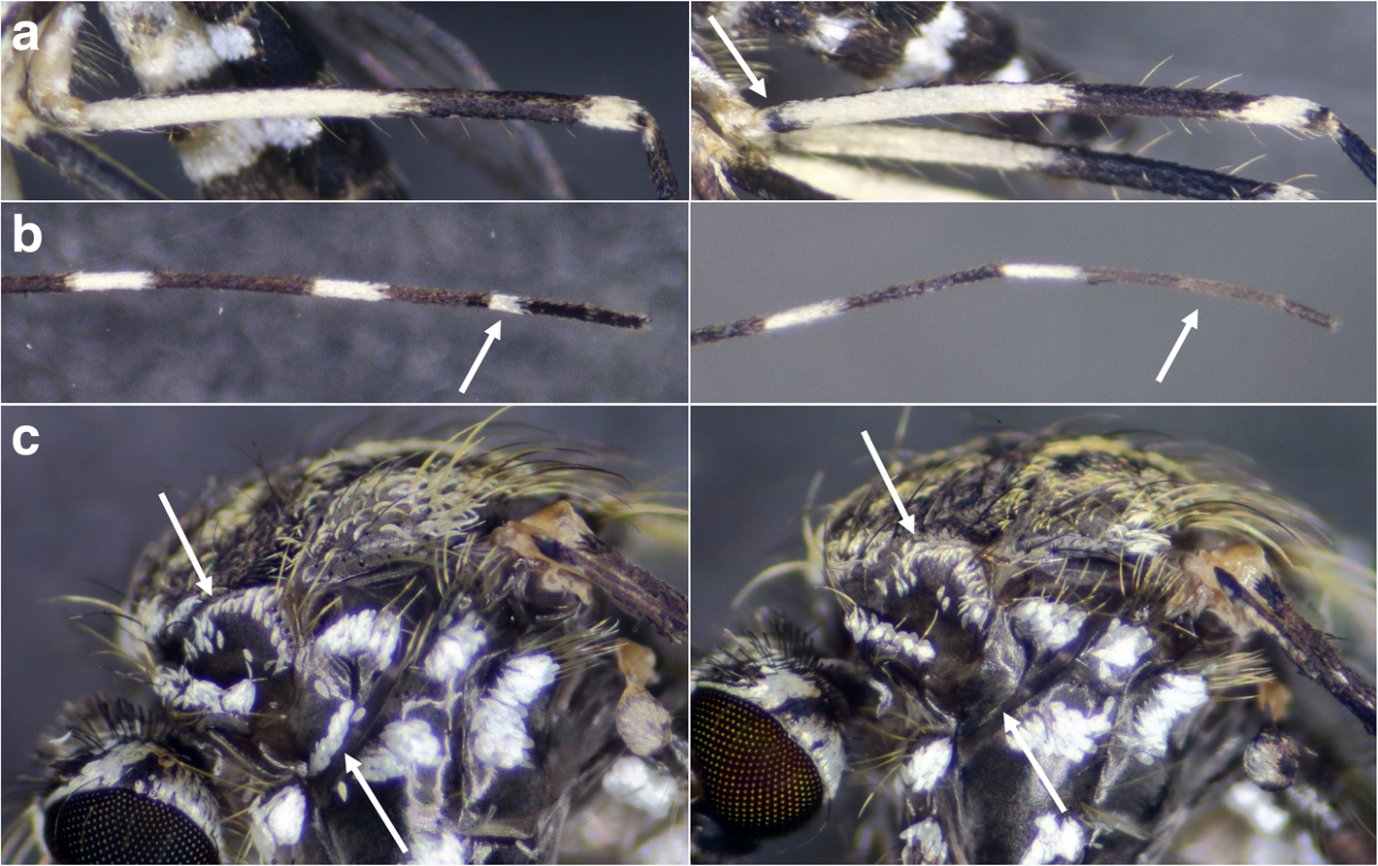
Diagnostic characteristics of *Ae. koreicus* females (left) in comparison to *Ae. j. japonicus* (right). **a** Hind femur. The arrow shows the dark subbasal band in *Ae. j. japonicus*, which is missing in *Ae. koreicus*. **b** Hind tarsus with tarsomeres II-V. Arrows show hindtarsomere IV. **c** Lateral view of thorax. Arrows show the postpronotum and the subspiracular patch (missing in *Ae. j. japonicus*). The costa can also be seen

The results of the morphological study are shown in Table 2 and a detailed evaluation is given in Additional file 2: Table S2. The most reliable characteristic was the base of the hind femur, which was always completely pale-scaled in *Ae. koreicus* and showed a dark subbasal band in all examined females of *Ae. j. japonicus* (Fig. 2a). Furthermore, all *Ae. koreicus* had a complete pale basal ring on hind tarsomere IV, so far as it was recognizable. Hind tarsomere V was usually entirely dark or showed only a few pale scales or an incomplete ring; a complete ring could not be observed. In *Ae. j. japonicus*, hind tarsomere IV was completely dark or bore only a few pale scales or an incomplete ring, while hind tarsomere V was always completely dark (Fig. 2b).

**Table 2:** Results of the morphological study. The order follows the diagnostic value for species identification. Numbers in parentheses give the count of an observed characteristic per total number of examined characteristics (see Additional file 2 for a detailed evaluation)

Character	*Ae. koreicus*	*Ae. j. japonicus*	Diagnostic value for species identification
Hind femur	Base entirely pale-scaled (43/43)	Dark subbasal band (30/30)	Diagnostic character
Hind tarsomere IV	Complete pale basal ring (41/41)	Most often dark (17/29), few pale scales (6/29) or incomplete pale ring (6/29)	Very variable in *Ae. j. japonicus*
Subspiracular patch (L + R)	Usually present (81/86), size very variable if present, 1 to 28 scales, mean number and median 10	Usually absent (59/60), maximum 1 scale counted (1/60)	*Ae. koreicus* can lack this patch, size varies a lot
Pedicel scales	Usually more pale than dark scales (40/43)	Usually more dark than pale scales (28/30)	Not easily quantifiable, sometimes scales are evenly distributed in both species
Costa	Usually no pale scales present (36/43), maximum few pale scales present (7/43)	Usually with pale scales (29/30), only once entirely dark	Number of pale scales very variable in *Ae. j. japonicus*
Postpronotum	Often no dark scales present (25/43) or only few dark scales (16/43); clear pattern of dark scales only twice	Usually no dark scales present (29/30), maximum of a few dark scales present (1/30)	Usually no or only a few dark scales present in both species but pale scales more whitish and less crescent shaped scales counted in *Ae. koreicus*
Fork scales	Usually no pale scales present (40/43), maximum 3 pale scales counted	Usually some pale scales up to 16 present (19/30) or no pale scales present (11/30)	Not easily recognizable, mainly dark in both species
Hind tarsomere V	Most often dark (29/41), few pale scales (10/41) or incomplete pale ring (2/41)	Entirely dark (29/29)	Usually dark in both species
Antepronotum	Broad pale scales (43/43)	Broad pale scales (30/30)	No discrimination possible
Color of scutal stripes	All scales golden-yellow, prescutellar area usually with paler scales (37/43), never silver-white	All scales golden-yellow (29/29)	No diagnostic character
Length of anterior dorsocentral stripes on scutum	Stripes usually reach the middle of the scutum (40/42) or slightly shorter (2/42)	Stripes reach at least the middle of the scutum (4/29), usually longer (25/29)	No diagnostic character
Tergite pattern			
Basomedian patch	Usually present on segments II-VII (38/41), at least present on segments II-VI (3/41)	Present on segments II–V up to segments II-VII (20/28), only on segment II (1/28) or absent on all segments (7/28)	No diagnostic character, very variable in *Ae. j. japonicus*
Size of basomedian patch	Ranges from a few pale scales (11/41), over pale spots (14/41) to clearly visible bands (15/41); once the patch was fused with the basolateral patches	If present, usually only a few pale scales (20/21), maximum a pale spot present (1/21)	No diagnostic character
Basolateral patch	Present on segments II–VII (43/43)	Present on segments II–VII (30/30)	No discrimination possible
Tergit VIII	Basolateral patches usually fused (35/42)	Basolateral patches usually not fused (21/28)	No diagnostic character

Subspiracular scales were absent in almost all of the *Ae. j. japonicus* specimens except for one sample, which had one subspiracular scale on one side. In *Ae. koreicus*, the number of subspiracular scales was very variable and ranged from 0 to 28 (Fig. 2c), with a mean and median of 10. Usually, a clear subspiracular patch could be recognized. Individuals with no scales at all were all derived from the same ovitrap. The highest number of scales in the five specimens from this site was two.

Less reliable characters for species identification were the pedicel scales and the costa due to a slight interspecific overlap of characteristics. In the *Ae. koreicus* specimens examined, more pale than dark scales usually were present on the pedicel, while the converse was true in *Ae. j. japonicus*. The costa was usually entirely dark in *Ae. koreicus* and only some specimens showed a few pale scales at most. One *Ae. j. japonicus* also had an entirely dark costa, while the others had only a few pale scales or more. A clear ventrobasal pale mark was recognizable in only six individuals.

Characteristics that showed no or only marginal differences between the two species were the erect fork scales on the vertex, the scales on the antepronotum and postpronotum, hind tarsomere V, the color and length of the scutal stripes as well as the tergite pattern.

The lines of the scutal pattern (see Additional file 3: Figure S1a) were formed by golden-yellow scales in both species but the color was more intense in *Ae. j. japonicus*. In the prescutellar area the scales often became paler in *Ae. koreicus*. The anterior dorsocentral lines usually reached the middle of the scutum in *Ae. koreicus*, where the posterior dorsocentral lines bend outwards at the transverse suture [31]. They were only a little longer in *Ae. j. japonicus*. Scales were never silver-white as described from the Belgian population of *Ae. koreicus*.

The abdominal tergite pattern (see Additional file 3: Figure S1b) showed basolateral spots on segments II-VII in all specimens of both species. The basolateral spots on segment VIII were most often divided in *Ae. j. japonicus* and fused in *Ae. koreicus*. The basomedian spots usually had a greater extent in *Ae. koreicus* than in *Ae. j. japonicus*.

## Discussion

### Distribution

Together with the three newly discovered sites in the city of Wiesbaden in the routine surveillance program of the KABS in 2017, the area colonized by *Ae. koreicus* extends over at least 50 km^2^, while the area initially surveyed by ovitraps is about 15 km^2^ and is now completely colonized. The morphological identification was confirmed by molecular methods. It remains unknown how the introduction took place. Since *Ae. koreicus* females lay their desiccation-resistant eggs in containers like *Aedes* (*Stegomyia*) *albopictus* (Skuse) and *Ae. j. japonicus*, the introduction could be due to the transport of plants or used tires [32, 33]. In Wiesbaden there is no large industrial zone, so the introduction could have happened over the Rhine harbor or plant centers, for example. Additionally, it cannot be excluded that the initial introduction was at Frankfurt Airport, located only 20 km away from the city. In the Netherlands, an airport was proven as the point of entry of IMS [34]. Between the airport and the city of Wiesbaden, no sampling of mosquitoes was done as part of our study. The population could have established in Frankfurt some years ago and spread to the west. In the east and south of the populated area, cemeteries were surveyed in past years and were all negative for *Ae. koreicus* and most of them negative for *Ae. j. japonicus*. Furthermore, Kampen et al. [12] did not find *Ae. koreicus* further westwards when sampling cemetery vases and other breeding sites, but they did detect *Ae. j. japonicus* in the area of Wiesbaden.

The distribution of *Ae. koreicus* in Italy shows that the species not only spreads actively, but also seems to be transported passively by trade of goods and road vehicles to more remote areas. It is assumed that the expansion to the south and west is mainly due to dense road connections in these directions [8]. In Switzerland, *Ae. koreicus* is apparently also distributed by vehicle transport. In 2015, the species was detected on highway E35 in ovitraps for *Ae. albopictus* surveillance north of the Alps [35]. It is therefore possible that the species reaches Germany passively from the south, similarly to *Ae. albopictus* [36]. So far, no record of *Ae. koreicus* has been made in the surveillance program along the German highways coming from Switzerland [36] (A. Jöst, unpublished observations).

The findings of *Ae. koreicus* in Sochi on the Black Sea coast of Russia in 2013 [15], in Slovenia in 2013 [16] and in an urban area in southern Hungary in 2016 [18] show that there is potential for further introductions of the species either from its native range or *via* dispersal inside Europe. In Germany, a single female from the southern part of the country was submitted to the citizen science project ‘Mueckenatlas’ in 2015 [17]. In a screening of this area, no further specimens or other IMS like *Ae. j. japonicus* were found. Our survey therefore represents the first record of an established population of *Ae. koreicus* in Germany.

The monthly mean temperatures in Wiesbaden are similar to the initially colonized area in north-eastern Italy. Even if the annual precipitation is lower in Wiesbaden, *Ae. koreicus* should find suitable conditions for a similar spread in Germany, which is shown by the distinct increase in numbers and expansion from one year to another. Additionally, *Ae. j. japonicus* showed a massive expansion in the KABS area and an increase in positive sites in 2017. This could be due to ideal conditions in this year for both species and a generally higher abundance, which facilitated colonization and therefore detection in cemetery vases. The precipitation in Wiesbaden, especially in July, was very high and monthly mean temperatures ranged around 20 °C from June to August [37].

The coexistence of *Ae. koreicus* and *Ae. j. japonicus* in Europe until now has only been reported from Ticino in Switzerland (E. Flacio, personal communication) and from Slovenia [16], but no detailed information is yet available. In Slovenia, *Ae. koreicus* was found as larvae only in 2013 among samples of *Ae. j. japonicus*. In 2014 and 2015, only *Ae. j. japonicus* could be found. It seems to be unlikely that *Ae. j. japonicus* displaced *Ae. koreicus* because both species are reported to occur sympatrically in Korea, colonizing the same breeding sites, with *Ae. koreicus* having a better adaptation to urban areas [1, 38]. In Italy, spatio-temporal annidation of *Ae. koreicus* and *Ae. albopictus* was observed [8, 39], which is also conceivable for *Ae. koreicus* and *Ae. j. japonicus* in Germany.

*Aedes koreicus* could not only be found in suburban areas with a high number of gardens and rural areas, but also frequently in and near forests. Similar results were observed by Baldacchino et al. [39], who caught more *Ae. koreicus* in forested than in urban areas with gravid traps which should reveal the preferred breeding site habitats, as with ovitraps. This shows that the species is not only restricted to the townships but can also colonize natural habitats.

### Morphological characterization and presumptive origin

The population of *Ae. koreicus* in Belgium was described as the morphological variant from the Korean island Jeju-do [5]. The authors performed an elaborate study with material from the Smithsonian Institute, comparing specimens from the Korean peninsula, the Jeju-do island and the Belgian population with the descriptions by Edwards [31] and Tanaka et al. [1]. In Wiesbaden, the characteristics of the Jeju-do and the Belgian populations could not be found but the individuals corresponded to the descriptions of the Korean mainland population.

In the individuals examined from the new population in Wiesbaden, the pedicel scales were mainly pale, while in Belgium they showed a characteristic pattern of dorsal and lateral pale spots. The erect fork scales on the vertex were usually completely dark, which is also described from the Korean mainland. The scales on the antepronotum were all broad and pale, while in Belgian and Jeju-do individuals crescent-shaped scales were also present. The main difference between the two forms is found in the basal pale bands of the hind tarsus [5]. The individuals in Wiesbaden never showed a complete ring on hind tarsomere V, which is the characteristic of the Belgian and the Jeju-do populations. It was usually entirely dark and sometimes bore some pale scales or an incomplete ring, which is also described for the species by Tanaka et al. [1].

This leads us to the conclusion that the newly emerged population of *Ae. koreicus* in Germany was not introduced from the Belgian or Italian/Swiss populations but originates from an independent introduction, most likely from the Korean peninsula and not from Jeju-do island. In this case, this is the first evidence for multiple introductions of this species to Europe. It is also possible that the Italian, Russian, Slovenian, German and Hungarian introductions were independent of the Belgian population, while the Swiss population very likely descends from the Italian one [10]. There is also evidence for multiple introductions of *Ae. j. japonicus* to North America [40] and Europe [11, 41], so this can also be assumed for *Ae. koreicus*. By characterizing the German individuals as a different morphological variant, derivation from the Belgian or Italian/Swiss populations can be excluded. This is also the first report of the morphological variant of *Ae. koreicus* from the Korean mainland outside of its native range. In European Russia and Hungary, the collected specimens were not characterized morphologically [15, 18] and in Slovenia only larvae were examined which cannot be attributed to one of the two forms [16].

### Discrimination of *Ae. koreicus* and *Ae. j. japonicus*

*Aedes koreicus* could clearly be identified and distinguished from *Ae. j. japonicus* morphologically, which is widely distributed and abundant in south-western Germany. Even though there is a high intraspecific variability in some of the examined characteristics, the morphological comparison revealed less variable and only slightly overlapping characteristics that allow an unambiguous identification. The main diagnostic characters for the distinction of the two species were the base of the hind femur, the pale basal scales on hind tarsomere IV and the subspiracular patch.

The best characteristic of these was the coloration of the hind femur. The base of the hind femur was completely pale-scaled in *Ae. koreicus*, while all individuals of *Ae. j. japonicus* showed a dark subbasal band (Fig. 2a). Only the so far non-invasive subspecies *Ae. japonicus yaeyamensis* is described without such a band and in *Ae. j. japonicus* only 1% of individuals examined in Japan and Korea lack a band [1].

All individuals of *Ae. koreicus* in our study had a complete pale basal ring on hind tarsomere IV (Fig. 2b). Hind tarsomere V was usually entirely dark and very rarely showed, at most, an incomplete ring. In *Ae. j. japonicus*, a complete ring was not observed on hind tarsomeres IV and V in our study. However, during a previous monitoring one specimen was found with a complete ring on hind tarsomere IV and some pale scales on hind tarsomere V, but all other examined characteristics clearly indicated that this individual was *Ae. j. japonicus*. The species seems to be very variable in this character, so that pale scales on hind tarsomeres IV and V cannot be used as the only feature for the determination of *Ae. koreicus*. Also, the other specific characteristics have to be examined for valid identification.

The subspiracular patch is also not a reliable character for identification, even if *Ae. koreicus* usually shows a clearly recognizable patch while in *Ae. j. japonicus* these scales most often are missing (Fig. 2c). This is because *Ae. koreicus* can also lack these scales, as was the case in one location where the maximum number of scales counted was two. Although it is possible that the patch was abraded by mechanical influence, it is not very likely. Adults were hatched from larvae and frozen shortly after emergence so they remained intact. Furthermore, other scales were not worn off and the scales in this part of the thorax are commonly well protected and preserved.

Other characteristics described by Tanaka et al. [1] were not unambiguously interpretable but supported the identification when typically shaped. The first of these were the pedicel scales, which were usually dominated by pale scales in *Ae. koreicus*, but not easily quantifiable. The ventrobasal mark on the costa was not always distinct in *Ae. j. japonicus* and *Ae. koreicus* also sometimes bore at least a few pale scales. On the postpronotum, often no dark scales could be observed in *Ae. koreicus*, which does not fit the description by Tanaka et al. [1]; on the other hand, the pale scales were usually more whitish than in *Ae. j. japonicus* and fewer crescent-shaped or thin scales were counted (Fig. 2c).

In the report by the ECDC [30], the anterior dorsocentral lines on the scutum of *Ae. koreicus* were described as very short in contrast to *Ae. j. japonicus*. This could not be observed in the Wiesbaden population, where these stripes were almost as long as in *Ae. j. japonicus* (Additional file 3: Figure S1a).

The identification of larvae could easily be achieved with the characteristics described by Tanaka et al. [1] and Versteirt et al. [5]. Where the pecten teeth are evenly spaced in *Ae. koreicus*, *Ae. j. japonicus* has one or two detached simple pecten teeth. The apical spines on the saddle are simple in *Ae. j. japonicus* and of a complex form in *Ae. koreicus* (see Additional file 4: Figure S2).

Tanaka et al. [1] reported a high degree of variability of the specific characteristics of females in both species, with an overlapping of these characteristics. It could be assumed that colonization of a new area by only a few individuals could have led to a lower variability as a result of a founder effect [42]. Instead, in this study *Ae. koreicus* showed a comparable variability in morphology to the population in Korea, with only slightly different dominating characteristics. Moreover, *Ae. j. japonicus* even shows a similar variability with similar distribution of characteristics.

## Conclusions

The arrival and spread of *Ae. koreicus* in Europe shows the need for thorough identification of detected individuals. This can be easily achieved by morphological determination of larvae and females if the specimens are in good condition. Even the discrimination to *Ae. j. japonicus* can be done without problems, despite the occasional overlap of the specific characteristics. In dubious cases molecular tools can clarify the assignment [4]. An ambiguous determination might carry the risk of an IMS remaining undiscovered [16, 17]. Since presently there is no common name for *Ae. koreicus*, and the species occurs together with the closely related “Asian bush mosquito” *Ae. j. japonicus* in Europe, we suggest changing the common name of *Ae. japonicus* to “Japanese bush mosquito” and introduce “Korean bush mosquito” for *Ae. koreicus*, following the scientific names.

## Additional files

Additional file 1: **Table S1.** Results of the ovitrap sampling. Numbers of collected eggs and larvae of the three species found per site and date. (PDF 256 kb)

Additional file 2: **Table S2.** Results of the morphological study. Examined features with numbers of observed characteristics of *Ae. koreicus* and *Ae. j. japonicus* females. (PDF 276 kb)

Additional file 3: **Figure S1.** Dorsal view of the scutum (**a**) and abdomen (**b**) of *Ae. koreicus.* The arrow shows the anterior dorsocentral line. (TIF 4283 kb)

Additional file 4: **Figure S2.** Larval characteristics of *Ae. koreicus* (left) and *Ae. j. japonicus* (right). **a** Pecten. **b** Apical spines on saddle. (TIF 2898 kb)

## Abbreviations

IMS: Invasive mosquito species
*nad*4: NADH dehydrogenase subunit 4
PCR: Polymerase chain reaction
