# Supplementary material for: First record and morphological characterization of an established population of Aedes (Hulecoeteomyia) koreicus (Diptera: Culicidae) in Germany
Source: Parasit Vectors. 2018 Dec 17;11:662. doi: 10.1186/s13071-018-3199-4 (PMC6296035; doi:10.1186/s13071-018-3199-4)
Supplement: Supplementary file 1 — Table S1. Results of the ovitrap sampling. Numbers of collected eggs and larvae of the three species found per site and date. (PDF 256 kb) [file 13071_2018_3199_MOESM1_ESM.pdf]

Site no.	Category	Species	10-Aug-2017	30-Aug-2017	20-Oct-2017
**1**	Sonnenberg cemetery	*Ae. koreicus*	121	29	8
		Ae. j. japonicus	1		
		Cx. pipiens/torrentium	80	18	
**2**	Field/garden	*Ae. koreicus*	3	6	
		Ae. j. japonicus			
		Cx. pipiens/torrentium	264	50	
**3**	Forest	*Ae. koreicus*	131		
		Ae. j. japonicus			
		Cx. pipiens/torrentium	73	2	
**4**	Forest	*Ae. koreicus*		44	58
		Ae. j. japonicus		32	24
		Cx. pipiens/torrentium		13	
**5**	Field/garden	*Ae. koreicus*	11	21	
		Ae. j. japonicus			
		Cx. pipiens/torrentium	114	6	
**6**	Forest	*Ae. koreicus*		85	
		Ae. j. japonicus	2	43	
		Cx. pipiens/torrentium	33	42	
**7**	Field/garden	*Ae. koreicus*	37	35	
		Ae. j. japonicus	35	43	
		Cx. pipiens/torrentium	62		
**8**	Bierstadt cemetery	*Ae. koreicus*	76	26	
		Ae. j. japonicus			
		Cx. pipiens/torrentium	158	40	32
**9**	Industrial area	*Ae. koreicus*	104		
		Ae. j. japonicus			
		Cx. pipiens/torrentium	66		
