# Supplementary material for: First record and morphological characterization of an established population of Aedes (Hulecoeteomyia) koreicus (Diptera: Culicidae) in Germany
Source: Parasit Vectors. 2018 Dec 17;11:662. doi: 10.1186/s13071-018-3199-4 (PMC6296035; doi:10.1186/s13071-018-3199-4)
Supplement: Supplementary file 2 — Table S2. Results of the morphological study. Examined features with numbers of observed characteristics of Ae. koreicus and Ae. j. japonicus females. (PDF 276 kb) [file 13071_2018_3199_MOESM2_ESM.pdf]

Characteristic	*Ae. koreicus*	*Ae. j. japonicus*
**No. of pedicel scales**	n=43	n=30
Less dark than pale scales	40	1
No. of dark and pale scales similar	3	1
More dark than pale scales	0	28
**No. of pale fork scales**	n=43	n=30
0	40	11
1-5	3	14
6-10	0	4
11-16	0	1
**Antepronotum**	n=43	n=30
Few dark scales	0	0
Broad pale scales	43	30
Pale falcate scales	0	0
**Postpronotum**	n=43	n=30
No dark scales	25	29
Few dark scales	16	1
Dark scales present	2	0
**Subspiracular patch, no. of scales**	n=86	n=60
0	5	59
1-5	12	1
6-10	29	0
11-15	21	0
16-20	13	0
21-25	4	0
26-30	2	0
**Costa**	n=43	n=30
Entirely dark	36	1
Few pale scales	7	12
Pale scales present, no mark	0	11
Ventrobasal pale mark	0	6
**Hindfemur**	n=43	n=30
Dark subbasal band	0	30
Entirely pale basally	43	0
**Hindtarsomere IV**	n=43	n=30
Entirely dark	0	17
Few pale scales	0	6
Pale basal ring incomplete	0	6
Pale basal ring complete	41	0
No result	2	1
**Hindtarsomere V**	n=43	n=30
Entirely dark	29	29
Few pale scales	10	0
Pale basal ring incomplete	2	0
Pale basal ring complete	0	0
No result	2	1
**Scutum, color of stripes**	n=43	n=30
All golden-yellow	6	29
Golden-yellow, prescutellar area white	37	0
Silver-white	0	0
No result	0	1
**Scutum, length of submedian stripe**	n=43	n=30
3/4 of anterior half of scutum	2	0
1/1 of anterior half of scutum	31	4
> 1/1 of anterior half of scutum	9	25
No result	1	1

**Tergite pattern**	***Ae. koreicus***	***Ae. j. japonicus***
**Basomedian patch present on segments**	n=43	n=30
No patch	0	7
II	0	1
II-V	0	7
II-VI	3	7
II-VII	38	6
No result	2	2
**Size of basomedian patch**	n=43	n=30
No pale scales	0	7
Few pale scales	11	20
Spots	14	1
Bands	15	0
Fused with bl spots	1	0
No result	2	2
**Basolateral patches present on segments**	n=43	n=30
II-VII	43	30
No result	0	0
**Tergit VIII**	n=43	n=30
Basolateral patches	7	21
Fused band	35	7
No result	1	2
