# Supplementary figures and images for: First record and morphological characterization of an established population of Aedes (Hulecoeteomyia) koreicus (Diptera: Culicidae) in Germany
Source: Parasit Vectors. 2018 Dec 17;11:662. doi: 10.1186/s13071-018-3199-4 (PMC6296035; doi:10.1186/s13071-018-3199-4)

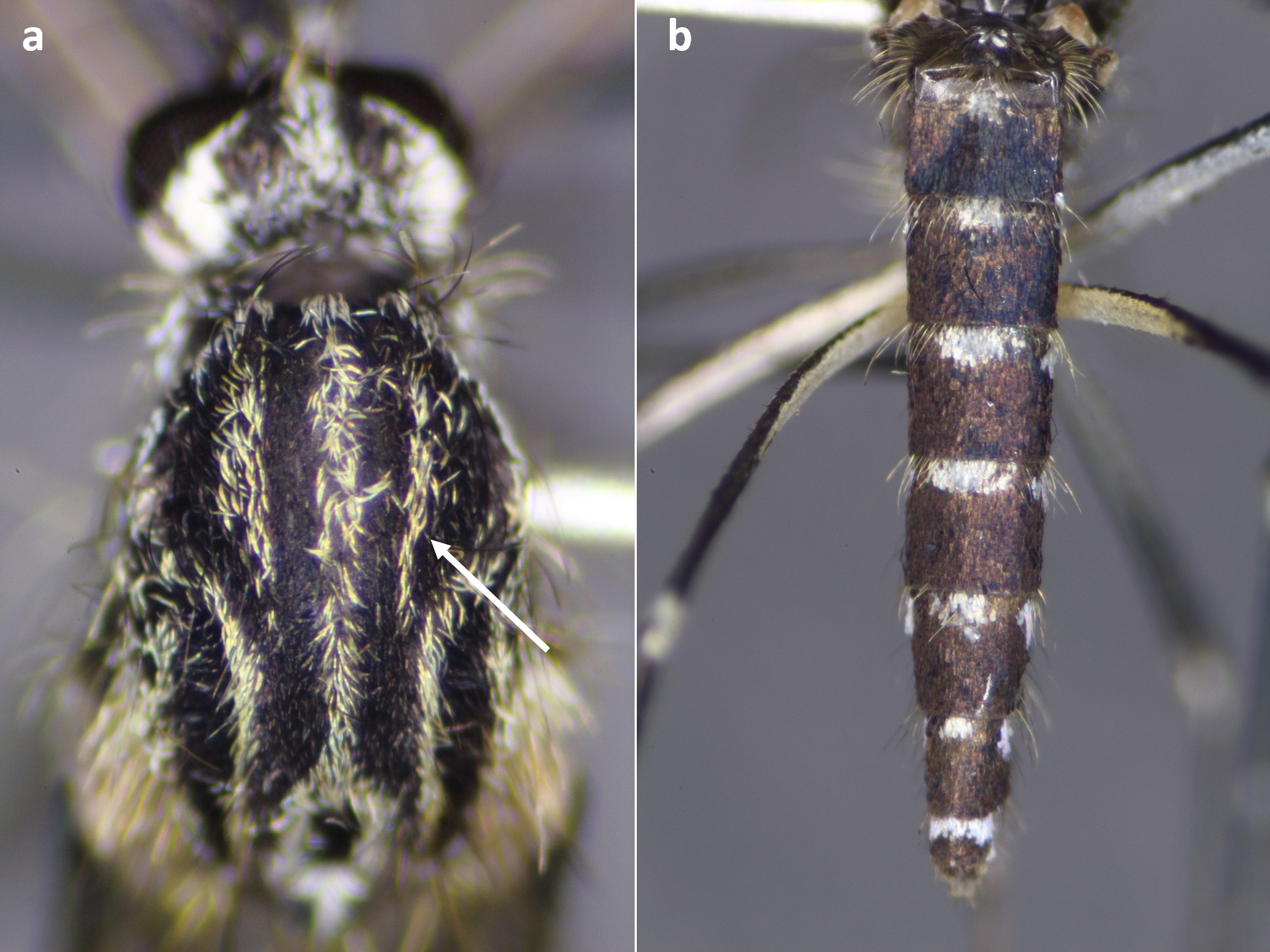

Supplement: Supplementary file 3 — Figure S1. Dorsal view of the scutum (a) and abdomen (b) of Ae. koreicus. The arrow shows the anterior dorsocentral line. (TIF 4283 kb) [file 13071_2018_3199_MOESM3_ESM.tif]

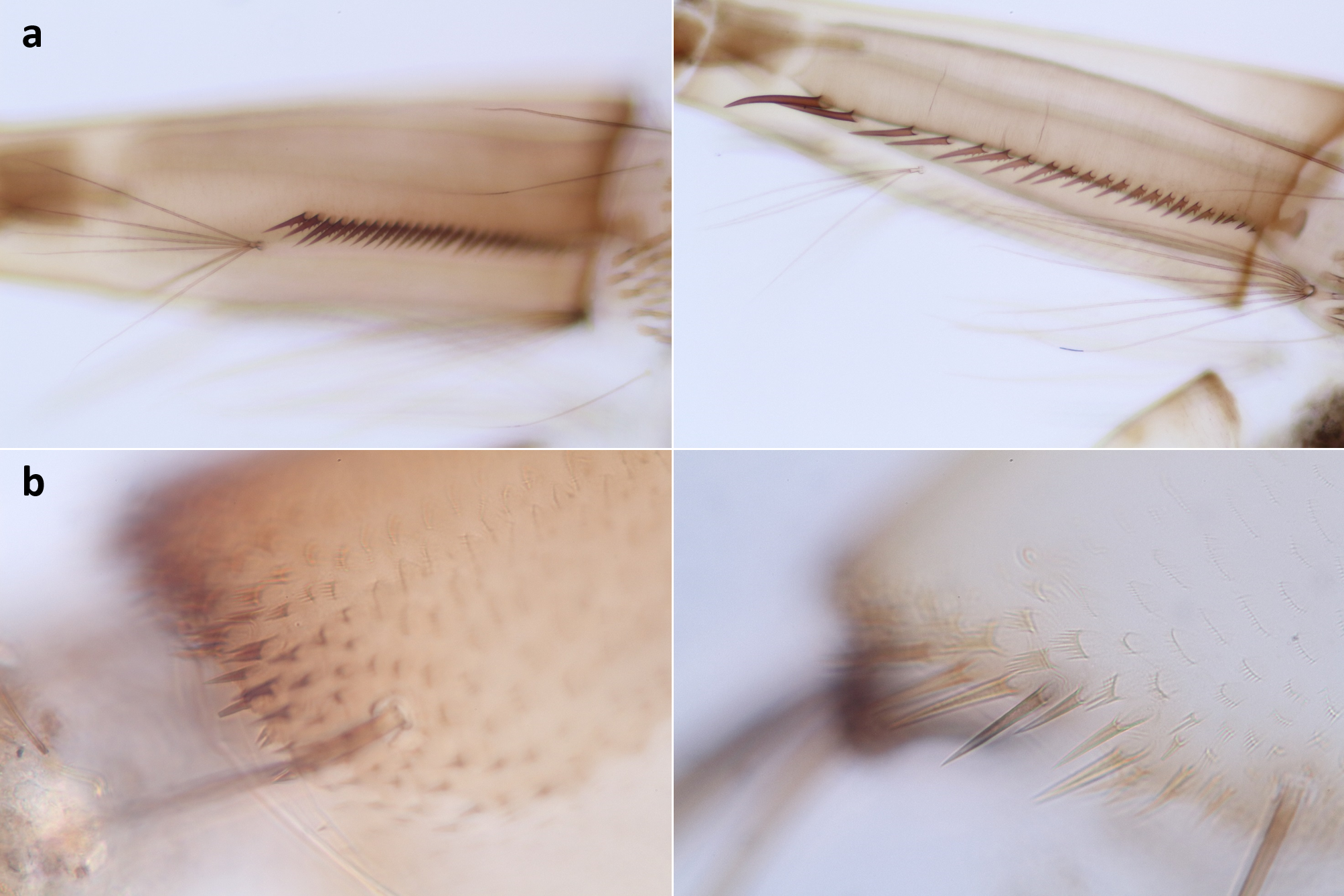

Supplement: Supplementary file 4 — Figure S2. Larval characteristics of Ae. koreicus (left) and Ae. j. japonicus (right). a Pecten. b Apical spines on saddle. (TIF 2898 kb) [file 13071_2018_3199_MOESM4_ESM.tif]
